# Legume lectin phytohemagglutinin reduces transepithelial electrical resistance by counteracting the chaperone function of heat shock protein-70

**DOI:** 10.1017/jns.2025.10017

**Published:** 2025-06-26

**Authors:** Karol Dokladny, Prashanth Setty, Pope L. Moseley, Henry C. Lin

**Affiliations:** 1 Department of Medicine, University of New Mexico, Albuquerque, NM, USA; 2 Siemens Healthcare Molecular Diagnostics, Berkeley, CA, USA; 3 Novo Nordisk Foundation Center for Protein Research, Faculty of Health and Medical Sciences, University of Copenhagen, Copenhagen, Denmark; 4 Section of Gastroenterology, New Mexico Veteran Affairs Health Care System, Albuquerque, NM, USA

**Keywords:** HSF1, HSP70, Lectins, Phytohemagglutinin (PHA), Protein folding, Ad70,heat shock protein 70 overexpressing adenovirus, AdTrack,control adenovirus, Caco-2,cancer coli-2, Cat.,catalogue, cDNA,complementary deoxyribonucleic acid, CHIP,carboxy terminus of heat shock protein 70-interacting protein, CO2,carbon dioxide, CT,cycle threshold, DAPI,4’,6-diamidino-2-phenyindole, DNA,deoxyribonucleic acid, EDTA,ethylenediaminetetraacetic acid, ELISA,enzyme-linked immunosorbent assay, FITC,fluorescein-5-isothiocyanate, HOP,heat shock protein 70-heat shock protein 90 organising protein, HRP,horseradish peroxidase, HSC,heat shock cognate, HSF,heat shock factor, HSP,heat shock protein, IG,immunoglobulins, IL,interleukin, kDa,kilodaltons, KFERQ,lysine-phenylalanine-glutamic acid-arginine-glutamine, LAMP,lysosomal-associated membrane protein, NaCl,sodium chloride, NaF,sodium fluoride, PBS,phosphate-buffered saline, PCR,polymerase chain reaction, pGL3,luciferase reporter vector, PHA,phytohemagglutinin, PMSF,phenylmethylsulfonyl fluoride, QPCR,quantitative polymerase chain reaction, RAW,reticuloendothelial anti-cancer agent, RNA,ribonucleic acid, RPM,revolutions per minute, RRKB,raw red kidney beans, rRNA,ribosomal ribonucleic acid, SD,standard deviation, SDS-PAGE,sodium dodecyl sulfate polyacrylamide gel electrophoresis, TBS,tris-buffered saline, TEER,transepithelial electrical resistance, TNF,tumour necrosis factor, Tris-HCl,tris(hydroxymethyl)aminomethane hydrochloride, Tween 20,polyoxyethylene (20) sorbitan monolaurate, ZO-1,zonula occludens-1

## Abstract

Legume lectins represent a broad class of environmental toxicants that bind to cell surface glycoproteins. Raw red kidney beans (RRKB), a widely consumed common source of dietary protein, are rich in the lectin phytohemagglutinin (PHA). Consumption of improperly cooked (which may require overnight presoaking and boiling at least at 100°C for 45 min) red kidney beans causes severe gastrointestinal symptoms. Since the relationship between lectin toxicity and the cellular chaperone machinery remains unknown, the study aimed to determine the effects of heat-denatured PHA on epithelial barrier function and heat shock protein 70 (HSP70) expression and its function as a molecular chaperone in PHA-treated Caco-2 cells and animals. Twelve male Sprague-Dawley rats were randomised to an *ad libitum* diet of either standard rat chow or chow containing 26% crude red kidney beans. We measured HSP70 and heat shock factor 1 gene expressions in the small intestine and HSP70 protein expression in Caco-2 cells. In Caco-2 cells, luciferase activity was measured to investigate protein folding. Fluorescein-5-isothiocyanate (FITC)-labelled lectin was used to study its intracellular uptake by Caco-2 cells. PHA lectin reduced transepithelial electrical resistance in Caco-2 cells. FITC-labelled PHA entered Caco-2 cells within 3 h of treatment. PHA treatment significantly reduced HSP70 levels and luciferase activity in Caco-2 cells, which was prevented by HSP70 overexpression. In rats fed RRKB chow consisting of legume lectins, we found reduced levels of HSP70 and heat shock factor 1. These observations suggest that lectins counter the protective function of HSP70 on intestinal barrier function.

## Introduction

Legume lectins represent a class of poorly recognised environmental toxicants. These sugar-binding proteins are difficult to destroy. In a recent large-scale study, the conditions required to inactivate lectin varied significantly, ranging from boiling for 15 min for black quinoa to 12 h of presoaking followed by boiling for 45 min for pinto beans.^(1)^ Overnight soaking significantly reduced the boiling time to 30 min at 100°C (60 min for unsoaked beans) to destroy phytohemagglutinin (PHA) activity.^(2,3)^ Inadvertent exposure to these toxic sugar-binding proteins may occur when beans are consumed without adequate cooking, which may cause acute gastroenteritis characterised by abdominal pain, nausea, vomiting, and diarrhoea.^(4)^ However, little is known about the mechanism of the toxic effects of edible bean lectins. Most information on the cellular effects of legume lectin comes from ricin, the lectin of castor beans, which is not considered an edible legume.^(5)^ Ricin exerts its toxicity by preventing rRNA from binding to the protein elongation factor, resulting in the shutdown of protein synthesis.^(6)^ Whether or not lectins from edible beans also interfere with the function of newly formed proteins is not known.

PHA is a lectin of red kidney beans, a legume that is common in the Western diet. There are five different tetramer populations of PHA consisting of two polypeptide chains called E with erythroagglutinating and L with leucoagglutinating properties held together by noncovalent bonds.^(7,8)^ A variety of toxic effects has been reported with PHA, including impaired growth, reduced food intake, bacterial overgrowth, reduced intestinal brush border, injury to the intestinal mucosa, and alterations in general metabolism and the systemic immune system (thymus atrophy).^(8–13)^ Interestingly, germ-free animals do not show the similar changes seen in conventional animals.^(14,15)^ In recent studies, no evidence of the toxicity of PHA lectin in rats was demonstrated. However, the growth of animals was slow, and the nitrogen fixation capacity of intestinal mucosal cells was impaired. In rats exposed to chow supplemented with uncooked red kidney beans, we have shown that intestinal barrier function is mainly impaired.^(4)^ Additionally, we documented greater total bacterial load in the liver, the proximal, mid, distal small intestine, and colon, and body weight loss. However, no differences in histological appearance were documented between the raw red kidney beans (RRKB)-fed group vs. control animals. The intestinal mucosa was histologically intact, and no increase in inflammatory cell levels was shown in the RRKB-fed group when compared to controls.^(4)^

PHA has a broad modulatory effect on innate and adaptive immunity. In the peripheral circulation, lectins induce the production of antibodies, including immunoglobulins IgG, IgA, IgE, and IgM.^(16,17)^ Apart from B cell activation,^(18)^ subcutaneous injection of PHA induces T-cell mitogenesis and T-cell-mediated immunocompetence,^(19–21)^ which requires the presence of monocytes-derived interleukin-6 (IL-6).^(22)^ In the mouse macrophage cell line RAW264.7, PHA induced the secretion of tumour necrosis factor-α (TNF-α) and nitric oxide, indicating an increased inflammatory response.^(23)^ Dietary lectins augmented IL-4 and IL-13 from human basophils.^(24)^ The markers of cytotoxicity of immunocytes, the granzyme B, and perforin expression levels were elevated in tumour tissues of PHA-L-treated mice when compared with control mice.^(25)^

The inducible chaperone protein, heat shock protein 70 (HSP is 72 kDa in size in rodents but will be referred to as HSP70) contributes to the intestinal barrier function.^(26,27)^ A key function of HSP70 is to protect newly expressed proteins from misfolding.^(28)^ Previous studies have shown decreased levels of stress proteins (HSP90 and HSP70) in rat gut and heat-stressed Cancer coli-2 (Caco-2) cells treated with PHA.^(29)^ However, the relationship between lectin toxicity and the cellular chaperone machinery remains largely unknown. Given the relationship between lectin toxicity and protein elongation,^(30)^ we wondered whether lectins might exert their effects on chaperone activity.

## Materials and methods

### Animals

Twelve male Sprague-Dawley rats (Charles River Laboratories, Wilmington, MA, USA) were used. The sample size was determined to achieve statistical power and analysis. No inclusion or exclusion criteria were used. The rats were housed in individual cages in the animal care facility under controlled temperature and humidity with a 12-hour light/12-hour dark cycle. The animals were given free access to water and standard rat chow (Harlan TEKLAD, TEKLAD Rodent Diet 8604, Madison, WI, USA) during a 7-day acclimatisation period. This study was approved by the Institutional Animal Care and Use Committee of the New Mexico Veteran Affair Health Care System, Albuquerque, NM (the date of approval and the Approval Number are 2007 and the Institutional Animal Care and Use Committee#07; the University of New Mexico036/Veteran Affair Health Care System#08_Henry Lin_07). In the present studies, all methods were performed in accordance with the relevant guidelines and regulations. We also confirm that the study is reported in accordance with ARRIVE guidelines.

### Experimental design

Rats were randomised to a standard solid-pellet rat chow diet (Control, n = 6) or chow enriched with 26% raw red kidney beans (RRKB, n = 6).^(31)^ Red kidney beans were ground in a Waring blender (Waring Laboratory Science, Torrington, CT, USA), combined with ground standard rat chow, and formed into solid pellets. The test diets were fed *ad libitum* to rats for 15 d. Tissue specimens were collected during a non-survival surgery on day 15 of the test diet. Intestinal samples were collected from the mid-region of the small intestine and colon under 4% isoflurane general anaesthesia. The luminal contents were flushed with phosphate-buffered saline (PBS). Tissue samples were then collected in duplicate. One sample from each point of the collection was placed in RNA Later (Qiagen, Germantown, MD, USA) and stored at –80°C.

### Chemicals

Culture media and related reagents were purchased from Life Technologies (Gaithersburg, MD, USA) and Sigma-Aldrich (Sigma-Aldrich Inc., St Louis, MO, USA). Transwell permeable filters were purchased from Corning (Corning Inc., NY, USA). PHA (Cat. # L8629) and FITC-labelled PHA (Cat. # 101097996) were purchased from Sigma-Aldrich and VWR, respectively. Anti-HSP70 (Cat. # ADI-SPA-812-D) antibodies were purchased from StressGen Biotechnologies (Victoria, British Columbia, Canada). Anti-β-actin was purchased from Sigma-Aldrich (Cat. # A2228). Secondary antibodies anti-rabbit IgG, HRP-linked (Cat. # 7074) were purchased from Cell Signaling Technology. Secondary antibodies anti-mouse IgG, HRP-linked (Cat. # 7076) were purchased from Cell Signaling Technology. The HSP70 Enzyme-Linked Immunosorbent Assay (ELISA) kit (Cat# ADI-EKS-700B) was purchased from Enzo Life Sciences (Farmingdale, NY, USA). The samples were analysed at 450 nm on the microplate reader (ELX 800, BIO-TEK Instruments). The sources of other chemicals are noted below.

### RNA isolation and cDNA synthesis

Tissues were homogenised at 20 hertz for 60 s in a TissueLyzer II (QIAGEN GmbH, Hilden, Germany) in 180 µl of tissue lysis buffer using one 0.5 mm steel bead. Total RNA was extracted using RNeasy mini-Kit (QIAGEN GmbH, Hilden, Germany). cDNA was synthesised according to the kit manufacturer’s instructions (GeneAmp RNA PCR, Applied Biosystems, Grand Island, NY, USA). A mixture of random hexamers and oligo d(T) primers was used with multiscribe reverse transcriptase (Applied Biosystems, Grand Island, NY, USA). A Quantitative Polymerase Chain Reaction (QPCR) was performed using 100 ng of the synthesised cDNA as a template. The Polymerase Chain Reaction (PCR) amplification conditions are as follows: denaturation: 95^o^C for 15 min; denaturation: 95^o^C for 30 s, annealing: 60^o^C for 30 s, extension: 68^o^C for 45 s; 40 cycles.

### Quantification of HSP70 and HSF1

HSP70 and HSF1 genes were amplified using the primers listed in Table 1. Primers were purchased from Real Time primers, LLC (Elkins Park, PA, USA). The qPCR conditions were optimised for the different primers to achieve similar amplification efficiencies to compare different amplicons. Product specificity was tested by melting curves, and product size was visualised by electrophoresis on agarose gel. qPCR was performed using Quantitech SYBR Green PCR Kit (QIAGEN GmbH, Hilden, Germany) according to the manufacturer’s instruction based on a final volume of 25 μl that contains 0.5 µM of each primer using 100–150 ng of DNA as a template for each reaction. The thermal cycling conditions included the following: an initial denaturation step at 95°C for 15 min, 40 cycles of denaturation at 95°C for 30 s, annealing at 60°C for 30 s, an extension at 68°C for 45 s, and a plate read step. A melt curve analysis was then followed. The total qPCR amplification cycle threshold (CT) was used to represent the respective genes. CT values of HSF1 and HSP70 genes were normalised against CT values of the host housekeeping gene β-actin to generate a delta-delta CT value (relative CT value). Delta-delta CT values are inversely proportional to the total amount of gene expression so that a higher gene copy number is represented by a lower relative CT value.

**Table 1.** List of primers used

Primer description	Primer sequence 5’-3’	
β-actin	Forward	TCG TAC CAC TGG CAT TGT GAT GGA
	Reverse	ACC GCT CAT TGC CGA TAG TGA TGA
HSP70	Forward	GCA AGG CCA ACA AGA TCA CCA TCA
	Reverse	TCC TCT TTC TCA GCC AGC GTG TTA
HSF1	Forward	GGG AGA TGA CTA CAC GGA TG
	Reverse	TAG GAT TCC CTG GAV TAC CC

### Cell culture

Caco-2 cell line was purchased from the American Type Culture Collection (Manassas, VA, USA) and maintained in Dulbecco’s Modified Eagle Medium (supplemented with 2 mM glutamine, 100 U/ml penicillin, 100 μg/ml streptomycin, and 10% foetal calf serum in a humidified atmosphere containing 5% CO_2_. Glutamine, penicillin, and streptomycin were purchased from GIBCO-BRL (Grand Island, NY, USA). Foetal calf serum was purchased from Atlanta Biologicals (Lawrenceville, GA, USA). Stock cultures were maintained in 100 mm dishes and were subcultured every 3–4 d. Cultures for all assays were maintained in 60 mm tissue culture plates and grown on Transwell filters following procedures as previously described.^(28)^

### Effect of PHA on transepithelial electrical resistance in Caco-2 cells

Transepithelial electrical resistance (TEER) of filter-grown Caco-2 cells was measured using an epithelial voltameter (World Precision Instruments, Saratoga, FL).^(28)^ Cells in short-term cultures were used for testing when the monolayer reached a resistance of at least 800 Ω/cm^2^ by 3–4 weeks. To study the effect of PHA on TEER, Caco-2 cells were exposed to PHA at the dose of 0 (control) or 200 μg/ml, and resistance readings were recorded at different time intervals (0, 12, 24, and 48 h). To study the effect of denatured lectin on TEER in Caco-2 cells, lectin was boiled at 100°C for 2 h.

### Time course of entry of PHA into epithelial cells

Cellular localisation of lectin was assessed by immunofluorescent antibody labelling. Caco-2 monolayers grown on coverslips were exposed to lectin for increasing time points. At the end of the experimental period, Caco-2 monolayers were washed twice in cold PBS (4°C) and fixed with 2% paraformaldehyde for 20 min and mounted on microscope slides (Erie Scientific, Portsmouth, NH) using ProLong Gold Antifade Reagent within 4′,6-diamidino-2-phenyindole (DAPI) (Invitrogen Corp.). Immunolocalizations of lectin-FITC protein were visualised using a Nikon fluorescence microscope (Nikon, Garden City, NY, USA) equipped with a Hamamatsu digital camera (Hamamatsu Photonics, Hamamatsu, Japan). Images were processed with Wasabi software (Hamamatsu Photonics Deutschland, Herrsching, Germany).

### Quantification of HSP70 by ELISA

Caco-2 cells grown in culture as monolayers were treated with 0 or 200 μg/ml of PHA for 48 h. Cells were washed with cold PBS, and clear cell extract was prepared by lysing the cells in an extraction buffer supplemented with protease inhibitor cocktail tablets (Complete Mini, Roche Diagnostics GmbH, Germany). Total protein content was measured by Bradford reagent (Cat. # 5000201; Quick Start^TM^ from BIO-RAD). Clear cell extract was used for the quantification of HSP70 by ELISA. HSP70 ELISA analysis with each sample run in duplicates was performed per the kit manufacturer’s instruction (HSP70 ELISA kit – ADI-EKS-700B, Enzo Life Sciences, Farmingdale, USA) manual. The extraction buffer was supplemented with protease inhibitor cocktail tablets (Cat. # 11836170001; Complete Mini, Roche Diagnostics purchased from Sigma-Aldrich).

### Western blot analysis of HSP70

Western blot analysis was performed to study the effect of lectin on HSP protein expression.^(28)^ Briefly, Caco-2 monolayers were exposed to PHA at a dose of 0, 100, or 200 μg/ml for 48 h. At the end of the experimental period, cells were immediately rinsed with ice-cold PBS and lysed with lysis buffer (50 mM Tris·HCl, pH 7.5, 150 mM NaCl, 500 μM NaF, 2 mM EDTA, 100 μM vanadate, 100 μM PMSF, 1 μg/ml leupeptin, 1 μg/ml pepstatin A, 40 mM paranitrophenyl phosphate, 1 μg/ml aprotinin, and 1% Triton X-100) and scraped. The cell lysates were placed in microfuge tubes and centrifuged using Eppendorf Centrifuge 5425R at 10,000 rpm for 10 min to yield a clear lysate. A clear supernatant was collected and stored at –20°C for further processing. Protein measurement was performed using Bio-Rad Protein Assay kit (DC Protein Assay, Bio-Rad Laboratories, Cat. # 5000111), and absorbances were read at 750 nm using a microplate reader (ELX 800, BIO-TEK Instruments). Laemmli gel loading buffer (Cat. # 1610737) was added to the lysate containing 20 μg of protein and boiled for 7 min. The samples were loaded and separated on an SDS-PAGE gel (Cat. # XP04202BOX; Invitrogen). The separated proteins were transferred from the gel to the nitrocellulose membrane (Trans-Blot Transfer Medium, Nitrocellulose Membrane, Cat. # 1620115; Bio-Rad Laboratories) overnight in a cold room at 4°C. The membrane was transferred from the apparatus and incubated for 2 h in a blocking solution (5% dry milk from Bio-Rad Cat. # 1706404 in TBS-Tween 20 buffer (Tween 20, Cat. # P9416 from Sigma-Aldrich) at room temperature with mild agitation. Then, the membrane was incubated with appropriate primary antibodies (see Materials and Methods, Chemicals Section for antibody specifications). Anti-HSP70 antibodies were used at 1:1000 dilution, and anti-β-actin was used at 1:2000 dilutions in a blocking solution overnight in a cold room at 4°C. After being rinsed three times (each wash for 10 min) in TBS-Tween buffer, the membrane was incubated (1 hour at room temperature) in appropriate secondary antibodies (see Materials and Methods, Chemicals Section for antibody specifications). A membrane was rinsed three times in TBS-Tween buffer (10 min each rinse) and developed using the Santa Cruz Western Blotting Luminol Reagents (Cat. # sc-2048, Santa Cruz Biotechnology, Santa Cruz, CA) on the Kodak BioMax MS film (Fisher Scientific, Pittsburgh, PA) using a film processor (Konica SRX-101A).

### Effect of PHA and HSP70 on protein folding using Luciferase assay

Since proper protein folding of the enzyme luciferase is required for luminescence, reduced luciferase activity is seen with protein misfolding.^(32)^ Caco-2 cells were transfected with constructs driving luciferase (pGL3) and incubated for 24 h to allow for optimal luciferase expression. For reporter gene assays testing HSP70 gain-of-function, pGL3 vector was co-transfected with either the control virus (AdTrack) or the virus driving HSP70 protein expression (Ad70). Following incubation with PHA at the dose of 0 (control) or 200 μg/ml at 37^°^C for 48 h, cells were washed twice with 1 ml of ice-cold PBS, lysed, incubated at room temperature for 15 min, scraped, and placed in microfuge tubes. A clear lysate was then obtained by centrifuging at 13,000 rpm for 20 s. Twenty microliters of the supernatant were assayed for luciferase activity using the luciferase assay system (Promega, Madison, WI). Luciferase values were determined by a Lumat LB9501 luminometer (Berthold, Wildbad, Germany).

### Statistical analysis

To determine the sample size for TEER experiments in Caco-2 cells, we used an online Computing Two Means – Sample Size calculator at https://select-statistics.co.uk/calculators/. The confidence level was set at 95% and power at 80%. Based on our previous studies,^(4)^ we estimated the difference between the untreated and treated groups at 20 and the variance at 70. The estimated sample size was 3. To determine the sample size for experiments of gene expression, we used the preliminary data and set the difference between treated and untreated groups at 5 and variance at 9. The estimated sample size was 6. Non-parametric data were analysed using the non-parametric Wilcoxon rank-sum test, and figures were generated in the statistical package R or licensed GraphPad Prism 6. Data represent mean ± SD.

## Results

### PHA lectin reduced transepithelial electrical resistance (TEER) in Caco-2 cells

**Fig. 1.**
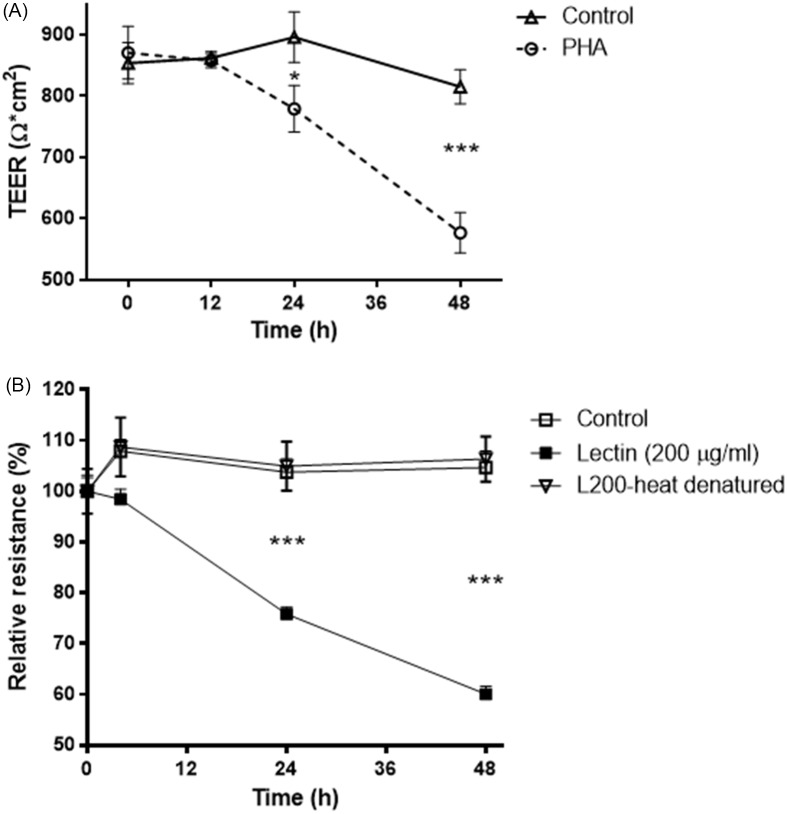
A: Time-course effect of legume lectin PHA (200 μg/ml) on transepithelial electrical resistance in Caco-2 cells. * P < 0.05; *** P < 0.001. Values are means ± SD. n = 3 per group. B: The effect of heat-denatured (100°C for 2 h) lectin PHA (200 μg/ml) on changes of transepithelial resistance in Caco-2 cells. *** P < 0.001. Values are means ± SD. n = 3 per group.

The effect of lectin on TEER was examined over 48 h (Fig. 1A). In control monolayers, there was a small (1–5% from the baseline) but not statistically significant increase when compared to the baseline in TEER at the early time points (for control monolayers, P = 0.7 baseline vs. 12-hour time point and P = 0.24 baseline vs. 24-hour time point). Although the causes of those increases remain unclear, they may be related to the fresh media added at time 0. When compared to TEER of control cells (854.3 ± 19.7) cultured in complete growth media, 200 μg/ml PHA treatment did not alter TEER (P = 0.68) after 12 h of treatment with PHA (873.3 ± 25.5). However, TEER of the PHA-treated cells dropped significantly in a time-dependent manner after 24 and 48 h, 773.6 ± 23.1 (P < 0.05) and 578.3 ± 15.4 (P < 0.0005), respectively. Since intense cooking is required to destroy legume lectins, we studied whether heat-denatured (100°C for 2 h) lectin PHA would affect transepithelial resistance in Caco-2 cells. Heat-denatured lectin PHA had no significant effect (P = 0.77; at 48-h time-point control monolayers vs. heat-denatured lectin-treated monolayers) on transepithelial resistance in Caco-2 cells, indicating that only intact protein can change the epithelial barrier integrity (Fig. 1B).

### Uptake of FITC-labelled PHA by Caco-2 cells

**Fig. 2.**
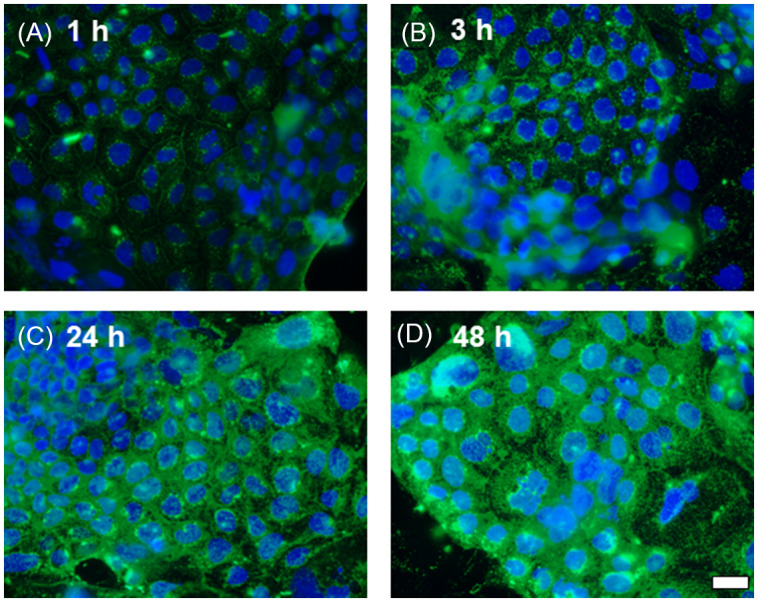
Time course of legume lectin entry into Caco-2 epithelial cells. Lectin-FITC protein (green). Nuclei were visualised with DAPI (blue). *A*: 1-hour exposure. *B*: 3-hour exposure. *C*: 24-hour exposure. *D*: 48-hour exposure. Scale bar 20 μm.

Caco-2 cells were exposed to PHA (200 µg/ml) conjugated with FITC across increasing time points. Uptake of FITC-labelled lectin by the cells was seen as soon as 1 hour after treatment (Fig. 2). During the initial time point, lectin fluorescence concentrated in the cytosol, and around the nucleus appeared as small dots. At the 3-hour time point, lectin intensity increased significantly in the cytoplasm and around the nucleus. Similarly, at 24- and 48-hour time points, lectin was present throughout the cytosol clustered around the nucleus. This data indicates that lectin enters the cell very quickly, and the process of entering the cells progresses over time.

### Lectin PHA reduced intracellular HSP70 levels in Caco-2 cells

**Fig. 3.**
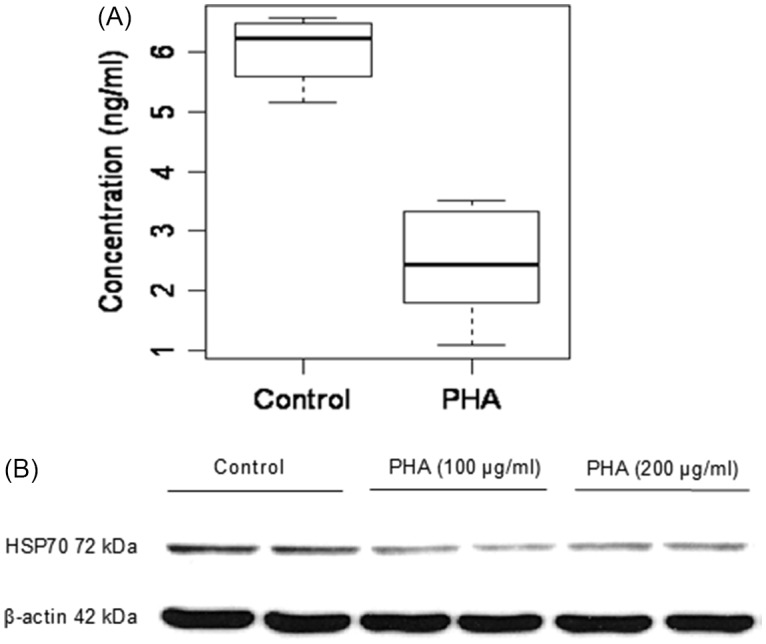
The effect of lectin PHA (200 μg/ml for 48 h) on HSP70 protein expression by ELISA (A) and Western blot analysis (B) in Caco-2 cells. A: Values are means ± SD (n = 3). P < 0.05 Control vs. PHA.

Protein estimation by an enzyme-linked immunosorbent assay (ELISA) on Caco-2 cell lysates treated with 200 μg/ml lectin PHA for 48 h showed a significant decrease (P < 0.05) of HSP70 levels (2.43 ± 0.95 ng/ml) compared to control cells grown in the absence of PHA (6.03 ± 0.56 ng/ml) (Fig. 3A). Western blot analysis of HSP70 levels in Caco-2 treated with lectin PHA (100 and 200 μg/ml for 48 h) confirmed these results (Fig. 3B).

### PHA-impaired protein folding in an HSP70-reversible fashion

**Fig. 4.**
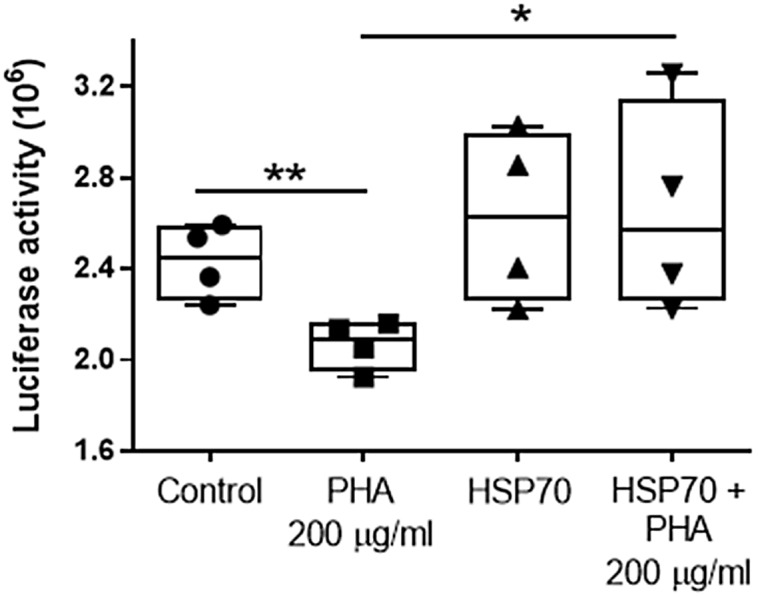
The effect of HSP70 on lectin PHA (200 µg/ml for 48 h)-induced protein folding by measuring the denaturation of luciferase in Caco-2 cells (decreased luciferase activity indicates greater protein misfolding). Values are means ± SD (n = 4). * P < 0.05; ** P < 0.01.

The effect of the lectin PHA (200 µg/ml) on protein folding was tested by measuring luciferase activity (decreased luciferase activity indicates greater protein misfolding). Cultures of Caco-2 cells were transfected with either pGL3 basic vector encoding the luciferase gene or co-transfected with constructs driving luciferase (pGL3) and HSP70 protein expression (Ad70). After a 24-hour incubation to allow for optimal luciferase expression, cells were subjected to PHA (200 µg/ml) treatment for 48 h following a measurement of luciferase activity. Controls were pGL3 single transfected cells. Compared to Controls (2.43 ± 0.16), the luciferase activity in Ad70 single transfected cells was not different (2.62 ± 0.38; P = 0.39 compared with control cells). Exposure to 200 μg/ml of PHA resulted in a significant decrease (2.07 ± 0.11; P < 0.01; compared with control cells) in luciferase activity in pGL3 single transfected cells, whereas in cells co-transfected with pGL3 and Ad70 (HSP70+PHA 200 μg/ml), this effect of PHA was abolished (2.66 ± 0.46; P = 0.4; compared with control cells. P < 0.05 compared with PHA-treated group). Thus, HSP70 overexpression inhibited the PHA-induced decrease in luciferase activity (Fig. 4). These data suggest that HSP70 exerts a protective effect against PHA-induced protein misfolding.

### PHA-containing raw red kidney beans decreased the expression of HSP70 and HSF1 in the small intestine

**Fig. 5.**
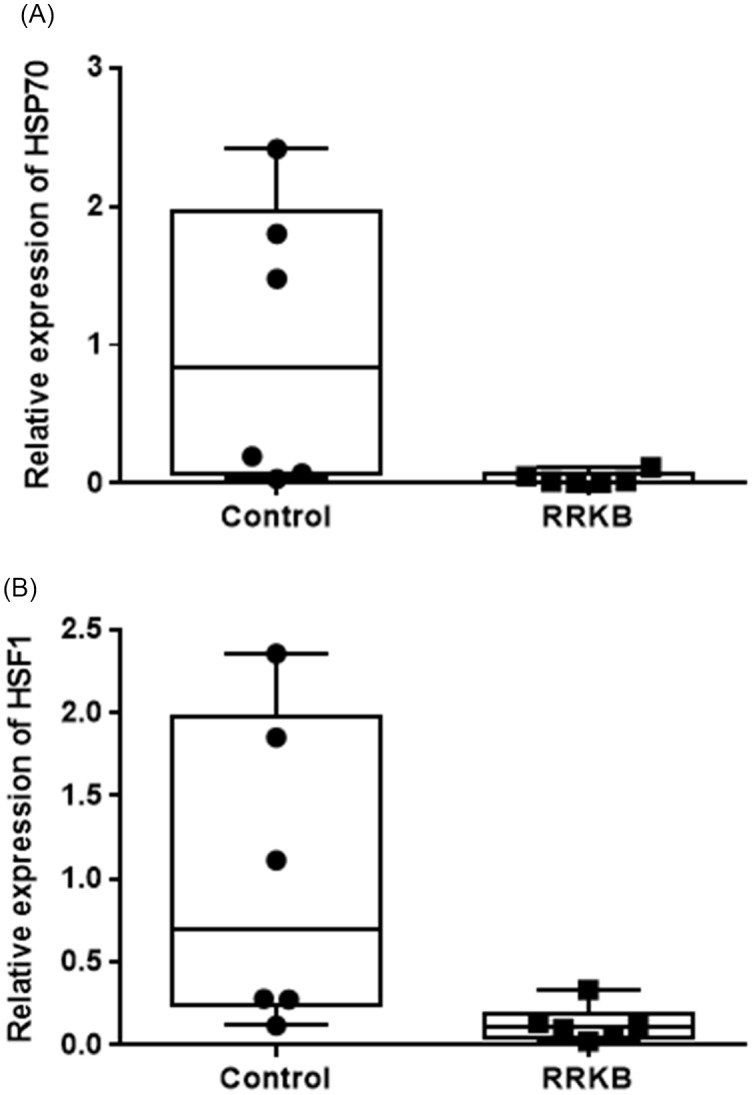
Effect of raw red kidney beans (RRKB) on HSP70 and HSF1 gene expression in the small intestine of rats. qPCR analysis was performed to calculate the expression levels of HSP70 (A) and HSF1 (B) genes in the small intestine of rats fed with RRKB. A statistically significant decreased expression of HSP70 was observed in the small intestine of RRKB-fed rats compared to control rats (P < 0.05) (Figure 5A). Similarly, a reduced level (P < 0.05) of HSF1 was observed in the small intestines of RRKB-fed rats. Relative values (2^–ΔΔCt^) are means ± SD. n = 6 per group.

To study the effect of raw red kidney beans containing lectins in native form, qPCR analysis was performed to calculate the expression levels of HSP70 and HSF1 genes in the small intestine and colon of rats fed with RRKB. A statistically significant decreased expression of HSP70 was observed in the small intestine of RRKB-fed rats compared to control rats (relative CT values: control; 1.0 ± 1.03, RRKB; 0.34 ± 0.04, P < 0.05) (Fig. 5A). However, no difference was seen in the colon (data not shown). Following these results, we then looked at the expression levels of the HSF1 gene in the small intestine and colon. A decreased level of HSF1 was observed in the small intestine of RRKB-fed rats (relative CT values: control; 1.0 ± 0.94, RRKB; 0.13 ± 0.11, P < 0.05) (Fig. 5B), but there was no difference in the colon (data not shown).

## Discussion

In this study, using Caco-2 cells, we demonstrate that PHA quickly enters gut epithelial cells, decreases Caco-2 epithelial barrier function, and reduces HSP and HSF1 (heat shock factor 1) levels in the small intestine. This reduced epithelial barrier function is associated with decreased HSP70 protein expression and impaired protein folding. Lectin PHA reduces HSP70 chaperone folding efficiency in Caco-2 cells. Heat-denatured lectin PHA did not affect the epithelial barrier function. The overexpression of HSP70 reverses the folding defect, suggesting that lectin-related disturbances in protein folding, which are important in lectin toxicity, are related to impaired cellular HSP70 accumulation and function. Taken together, these results implicate the lectin-induced impairment of cellular chaperone function in disrupting epithelial barrier function.

Lectins are unique carbohydrate-binding proteins that are found in many plants and are present in high concentrations in beans and other legumes.^(33,34)^ Phytohemagglutinin (PHA) is a lectin in the red kidney bean (Phaseolus vulgaris). Having a carbohydrate recognition domain, PHA binds to sugars expressed on the surface of epithelial cells and exerts cytotoxicity.^(35)^ The extremely toxic effects of ricin derived from castor beans (a lethal effect at a dose as small as 500 micrograms when this lectin is inhaled by humans; http://www.bt.cdc.gov/agent/ricin/facts.asp) are related to its ability to the cell membrane, internalisation into polarised epithelial cells by specific binding to yet unknown receptors, delivery to the trans-Golgi network in the cytosol, lysosomes, or nucleus, and subsequent inhibition of protein synthesis.^(5,36–38)^ Lectins, in contrast to mucoadhesive polymers, specifically recognise receptor-like molecules on cell membranes and bind directly to the epithelial cells rather than to the mucus layer. This receptor-mediated binding may promote the active transport of larger molecules through endocytosis.^(38)^ In mouse pituitary cells, wheat germ agglutinin-horseradish peroxidase binds to the cell surface membrane, followed by endocytosis, and targets the Golgi apparatus.^(39)^ Additionally, glycoproteins or glycolipids can serve as lectin-binding sites.^(40)^ In this study, we found that phytohemagglutinin entered the cell rapidly to cluster around the nucleus, and the effect increased in a time-dependent manner (Fig. 2). We also found that phytohemagglutinin reduced transepithelial electrical resistance in Caco-2 cells (Fig. 1A), which was prevented when cells were exposed to heat-denatured lectin (Fig. 1B). In our previous studies, this adverse effect on transepithelial electrical resistance persisted with increased duration of exposure to 72 h, and the transepithelial electrical resistance had failed to recover to the pre-treatment levels even after washing off PHA.^(4)^

Lectins are highly resistant to enzymatic proteolysis in the intestinal lumen and are capable of surviving heat treatment.^(41–44)^ The complete degradation of legume lectins may require severe cooking methods, such as boiling beans using a pressure cooker. Acute gastroenteritis is a well-recognised, short-term consequence of limited legume lectin exposure.^(41,45)^ This acute toxicity is thought to be the result of lectin binding to carbohydrate moieties on the gut epithelial surface, resulting in epithelial barrier dysfunction, maldigestion, and malabsorption.^(4,46,47)^ Another important feature of legume lectin-induced epithelial dysfunction is a loss of secretory control leading to diarrhoea.^(9,11,48)^ In the present study, we showed that the toxicity of the red kidney bean lectin PHA includes a reduced expression of HSP70 and HSF1 and interference with chaperone-mediated stabilisation of protein folding.

Interesting recent literature suggests that lectin-mediated cytotoxicity in cancer cells is caused by the activation of autophagic pathways.^(49)^ Autophagy is subdivided into 3 main subtypes: chaperone-mediated autophagy, microautophagy, and macroautophagy.^(50–52)^ In chaperone-mediated autophagy, cytosolic proteins with a characteristic pentapeptide motif (KFERQ - lysine-phenylalanine-glutamic acid-arginine-glutamine)^(53,54)^ are recognised by HSPA8/HSC70 (heat shock 70 kDa protein 8) and delivered to the lysosomes via HSC70 and cochaperones, including CHIP (HSC70-interacting protein), HSP40, and HOP (HSP70–HSP90 organising protein). After motif recognition by HSPA8 and subsequent binding to LAMP2A (lysosomal-associated membrane protein 2A), the target proteins are internalised into the lysosomes, where they are unfolded and eventually degraded. The finding that autophagy is controlled, at least in part, by HSP expression^(55)^ offers the possibility that the ability of lectins to disrupt HSP accumulation and function accounts for their observed ability to activate autophagic pathways.

The folding of newly formed proteins is facilitated by protein glycosylation in the endoplasmic reticulum.^(56)^ Inhibited glycosylation leads to protein misfolding and their clustering within the endoplasmic reticulum.^(57)^ Such clustered misfolded proteins attract and bind to a variety of molecular chaperones that have lectin-like activities and modulate the production of chaperones.^(58,59)^ We used the term “protein misfolding” to broadly describe a variety of intracellular events that destabilise proteins by hampering or distorting their ability to achieve their native conformation through unfolding, misfolding, and aggregation.^(60–62)^ To perform their function, proteins must be structurally active. Since a maximally stable protein is minimally active, proteins are intrinsically unstable. Such instability threatens the success of the initial folding of the protein to achieve its native conformational structure. Even when initial folding is successful, several physicochemical challenges, such as heat, have the effect of destabilising the native conformation of newly formed protein, leading to misfolding and intracellular protein aggregation.^(63)^ To survive, cells have evolved several protective mechanisms to promote proper protein folding and deal with proteins that have unfolded, misfolded, or aggregated, including the use of heat shock proteins. In the intestine, HSP70 plays a key role as the molecular chaperone of newly formed proteins.^(64–66)^ In this study, we found that overexpression of HSP70 reversed the protein folding impairment caused by lectin (Fig. 4). Greater availability of HSP70 may also facilitate the process of disaggregation of proteins.

In the present study, we have focused on HSP70 as this chaperone protein is the predominant^(67)^ and best-characterised member of the heat shock protein family. Specifically, HSP70 is critical for protecting cells against oxidative and heat-related injury,^(68)^ maintenance of epithelial barrier function,^(28,69)^ in part, by regulating the expression of occludin via heat shock factor-1.^(70)^ In addition, the availability of HSP70 is a key factor in cellular protection against apoptotic challenges,^(71,72)^ suppression of TNF-α mediated cellular injury,^(73,74)^ inhibition of endotoxin-induced shock and death^(71)^ through suppression of cytokine production.^(75–77)^

In the present study, we demonstrated that the red kidney bean lectin, phytohemagglutinin (PHA), rapidly entered the cell, reduced transepithelial electrical resistance in Caco-2, and interfered with protein folding. This toxic effect can be countered with the stress-related heat shock protein HSP70. The toxicity of this legume lectin could be explained by its ability to inhibit HSF1 gene expression and, in turn, the availability of HSP70. We extended the in-vitro observations by showing that exposure to plant lectin in the form of a raw red kidney bean-supplemented diet decreased HSP70 and HSF1 gene expression in rats. Our findings support the previous reports demonstrating that plant lectins inhibit intracellular HSP70 and HSP90 in the heat stress model.^(29)^

Beans represent a unique food category containing numerous beneficial nutrients, including proteins, complex carbohydrates, as well as vitamins, and minerals. They also contain potentially toxic lectins. Due to their heat resistance and imperviousness to digestive enzymes, plants containing toxic lectins are naturally protected against foraging mammals. Legumes containing foodstuffs can be consumed but only after prolonged soaking and extensive heat treatment to inactivate those toxic components.^(1,2)^ Prolonged aqueous boiling of beans may be sufficient for denaturing bean lectins, however, previous reports suggest that even after extended cooking or digestion in the gut, lectins may remain immunologic and exert biologic effects.^(3)^ As we gravitate towards natural foods, the possibility that chronic low-level exposures to toxic lectins may exert adverse effects on human health is a real concern. Lectin consumption has been openly speculated to be involved in the aetiology of many pathological conditions, including common food poisoning, rheumatoid arthritis, insulin-dependent diabetes, IgA nephropathy, and peptic ulcers.^(41)^ As our previous studies in animals suggested, common sucrose can be protective against potential lectin toxicity characterised by an increase in intestinal permeability, bacterial load, and translocation.^(4)^ Our present study demonstrates that boiling lectin is sufficient to deactivate its ability to disrupt the epithelial barrier. Future studies are needed to fully understand the cellular and molecular mechanisms leading to lectin-induced diminished cellular chaperone activity and its effect on tight junction proteins to develop safe interventions against lectin-induced toxicity.

The strength of our study is that it extends our knowledge of the cellular mechanisms of legume lectin toxicity. Our data show for the first time that lectin targeting heat shock proteins interferes with cellular chaperone activity, which can lead to impaired intestinal barrier function but can be rescued by overexpression of HSP70. Our findings support the injurious role of legume lectin and the protective role of HSP70 on intestinal barrier function. However, it is not known whether the findings in Caco-2 cells apply to other intestinal cell lines. Given that the epithelial barrier function is compromised by legume lectin exposure, it would be beneficial in future studies to examine the effect of PHA lectin on tight junction protein expression (occludin and ZO-1).

Since intense cooking is required to destroy legume lectins, our preference for colourful, natural-appearing foods rather than thoroughly cooked mush may lead to greater exposure to legume lectins. Exposure to these dietary toxicants is well known to cause gastrointestinal symptoms. In this study, we extended this understanding of this toxicity by showing the action of dietary lectins on protein expression and folding.

## Data Availability

Interested parties can contact Henry C. Lin, M.D. (helin@salud.unm.edu) or Karol Dokladny, Ph.D. (kdokladny@salud.unm.edu) to request access to the data.

## References

[1] Adamcova A , Laursen KH , Ballin NZ. Lectin activity in commonly consumed plant-based foods: calling for method harmonization and risk assessment. Foods. 2021;10(11):2796. 34829077 10.3390/foods10112796 PMC8618113

[2] Venter FS , Thiel PG. Red kidney beans--to eat or not to eat? S Afr Med J. 1995;85(4):250–252. 7777998

[3] Pusztai A , Grant G. Assessment of lectin inactivation by heat and digestion. Methods Mol Med. 1998;9:505–514. 21374488 10.1385/0-89603-396-1:505

[4] Ramadass B , Dokladny K , Moseley PL , Patel YR , Lin HC. Sucrose co-administration reduces the toxic effect of lectin on gut permeability and intestinal bacterial colonization. Dig Dis Sci. 2010;55(10):2778–2784. 20686845 10.1007/s10620-010-1359-2

[5] Spooner RA , Hart PJ , Cook JP , et al. Cytosolic chaperones influence the fate of a toxin dislocated from the endoplasmic reticulum. Proc Natl Acad Sci U S A. 2008;105(45):17408–17413. 18988734 10.1073/pnas.0809013105 PMC2580750

[6] May KL , Yan Q , Tumer NE. Targeting ricin to the ribosome. Toxicon. 2013;69:143–151. 23454625 10.1016/j.toxicon.2013.02.001 PMC3672271

[7] Nagae M , Soga K , Morita-Matsumoto K , et al. Phytohemagglutinin from Phaseolus vulgaris (PHA-E) displays a novel glycan recognition mode using a common legume lectin fold. Glycobiology. 2014;24(4):368–378. 24436051 10.1093/glycob/cwu004

[8] Banwell JG , Boldt DH , Meyers J , Weber FL, Jr. Phytohemagglutinin derived from red kidney bean (Phaseolus vulgaris): a cause for intestinal malabsorption associated with bacterial overgrowth in the rat. Gastroenterology. 1983;84(3):506–515. 6822324

[9] Banwell JG , Abramowsky CR , Weber F , Howard R , Boldt DH. Phytohemagglutinin-induced diarrheal disease. Dig Dis Sci. 1984;29(10):921–929. 6383746 10.1007/BF01312481

[10] Evans RJ , Pusztai A , Watt WB , Bauer DH. Isolation and properties of protein fractions from navy beans (Phaseolus vulgaris) which inhibit growth of rats. Biochim Biophys Acta. 1973;303(1):175–184. 4633880 10.1016/0005-2795(73)90159-1

[11] Wilson AB , King TP , Clarke EM , Pusztai A. Kidney bean (Phaseolus vulgaris) lectin-induced lesions in rat small intestine: 2. Microbiological studies. J Comp Pathol. 1980;90(4):597–602. 7024332 10.1016/0021-9975(80)90108-5

[12] Ceri H , Falkenberg-Anderson K , Fang RX , Costerton JW , Howard B , Banwell JG. Bacteria-lectin interactions in phytohemagglutinin-induced bacterial overgrowth of the small intestine. Can J Microbiol. 1988;34(8):1003–1008. 3208205 10.1139/m88-176

[13] Pusztai A , Clarke EM , Grant G , King TP. The toxicity of Phaseolus vulgaris lectins. Nitrogen balance and immunochemical studies. J Sci Food Agric. 1981;32(10):1037–1046. 7300257 10.1002/jsfa.2740321014

[14] Jayne-Williams DJ , Hewitt D. The relationship between the intestinal microflora and the effects of diets containing raw navy beans (Phaseolus vulgaris) on the growth of Japanese quail (Coturnix coturnix japonica). J Appl Bacteriol. 1972;35(2):331–344. 4558957 10.1111/j.1365-2672.1972.tb03705.x

[15] Jayne-Williams DJ , Burgess CD. Further observations on the toxicity of navy beans (Phaseolus vulgaris) for Japaneses quail (Coturnix coturnix japonica). J Appl Bacteriol. 1974;37(1):149–169. 4846736 10.1111/j.1365-2672.1974.tb00425.x

[16] Vojdani A , Afar D , Vojdani E. Reaction of lectin-specific antibody with human tissue: possible contributions to autoimmunity. J Immunol Res. 2020;2020:1438957. 32104714 10.1155/2020/1438957 PMC7036108

[17] Kumar S , Verma AK , Sharma A , et al. Phytohemagglutinins augment red kidney bean (Phaseolus vulgaris L.) induced allergic manifestations. J Proteomics. 2013;93:50–64. 23454658 10.1016/j.jprot.2013.02.003

[18] de Santana Brito J , Ferreira GRS , Klimczak E , et al. Lectin from inflorescences of ornamental crop Alpinia purpurata acts on immune cells to promote Th1 and Th17 responses, nitric oxide release, and lymphocyte activation. Biomed Pharmacother. 2017;94:865–872. 28810516 10.1016/j.biopha.2017.08.026

[19] Tella JL , Lemus JA , Carrete M , Blanco G. The PHA test reflects acquired T-cell mediated immunocompetence in birds. PLoS One. 2008;3(9):e3295. 18820730 10.1371/journal.pone.0003295 PMC2546448

[20] Maciel EV , Araujo-Filho VS , Nakazawa M , Gomes YM , Coelho LC , Correia MT. Mitogenic activity of Cratylia mollis lectin on human lymphocytes. Biologicals. 2004;32(1):57–60. 15026026 10.1016/j.biologicals.2003.12.001

[21] Maizel AL , Mehta SR , Hauft S , Franzini D , Lachman LB , Ford RJ. Human T lymphocyte/monocyte interaction in response to lectin: kinetics of entry into the S-phase. J Immunol. 1981;127(3):1058–1064. 6790609

[22] Ceuppens JL , Baroja ML , Lorre K , Van Damme J , Billiau A. Human T cell activation with phytohemagglutinin. The function of IL-6 as an accessory signal. J Immunol. 1988;141(11):3868–3874. 3263438

[23] Kim D , Yamasaki Y , Jiang Z , et al. Comparative study on modeccin- and phytohemagglutinin (PHA)-induced secretion of cytokines and nitric oxide (NO) in RAW264.7 cells. Acta Biochim Biophys Sin (Shanghai). 2011;43(1):52–60. 21148191 10.1093/abbs/gmq105

[24] Haas H , Falcone FH , Schramm G , et al. Dietary lectins can induce in vitro release of IL-4 and IL-13 from human basophils. Eur J Immunol. 1999;29(3):918–927. 10092096 10.1002/(SICI)1521-4141(199903)29:03<918::AID-IMMU918>3.0.CO;2-T

[25] Wang P , Leng X , Duan J , et al. Functional component isolated from phaseolus vulgaris lectin exerts in vitro and in vivo anti-tumor activity through potentiation of apoptosis and immunomodulation. Molecules. 2021;26(2):498. 33477737 10.3390/molecules26020498 PMC7832403

[26] Wu X , Zhang Y , Yin Y , et al. Roles of heat-shock protein 70 in protecting against intestinal mucosal damage. Front Biosci (Landmark Ed). 2013;18:356–365. 23276928 10.2741/4106

[27] Akagi R , Ohno M , Matsubara K , Fujimoto M , Nakai A , Inouye S. Glutamine protects intestinal barrier function of colon epithelial cells from ethanol by modulating Hsp70 expression. Pharmacology. 2013;91(1–2):104–111. 23328693 10.1159/000345930

[28] Dokladny K , Wharton W , Lobb R , Ma TY , Moseley PL. Induction of physiological thermotolerance in MDCK monolayers: contribution of heat shock protein 70. Cell Stress Chaperones. 2006;11(3):268–275. 17009600 10.1379/CSC-194R.1 PMC1576477

[29] Ovelgonne JH , Koninkx JF , Pusztai A , et al. Decreased levels of heat shock proteins in gut epithelial cells after exposure to plant lectins. Gut. 2000;46(5):679–687. 10764712 10.1136/gut.46.5.680 PMC1727920

[30] Olsnes S , Refsnes K , Pihl A. Mechanism of action of the toxic lectins abrin and ricin. Nature. 1974;249(458):627–631. 4857870 10.1038/249627a0

[31] Banwell JG , Howard R , Kabir I , Adrian TE , Diamond RH , Abramowsky C. Small intestinal growth caused by feeding red kidney bean phytohemagglutinin lectin to rats. Gastroenterology. 1993;104(6):1669–1677. 8500725 10.1016/0016-5085(93)90644-r

[32] Cashikar AG , Duennwald M , Lindquist SL. A chaperone pathway in protein disaggregation. Hsp26 alters the nature of protein aggregates to facilitate reactivation by Hsp104. J Biol Chem. 2005;280(25):23869–23875. 15845535 10.1074/jbc.M502854200 PMC1391974

[33] Boylan MT , Sussex IM. Purification of an endopeptidase involved with storage-protein degradation in phaseolus-vulgaris L cotyledons. Planta. 1987;170(3):343–352. 24232964 10.1007/BF00395026

[34] Rudiger H , Gabius HJ. Plant lectins: occurrence, biochemistry, functions and applications. Glycoconj J. 2001;18(8):589–613. 12376725 10.1023/a:1020687518999

[35] Sharon N , Lis H. The structural basis for carbohydrate recognition by lectins. Adv Exp Med Biol. 2001;491:1–16. 14533786 10.1007/978-1-4615-1267-7_1

[36] Gonatas NK , Avrameas S. Detection of carbohydrates with lectin-peroxidase conjugates. Methods Cell Biol. 1977;15:387–406. 327205

[37] van Deurs B , Sandvig K , Petersen OW , Olsnes S , Simons K , Griffiths G. Estimation of the amount of internalized ricin that reaches the trans-Golgi network. J Cell Biol. 1988;106(2):253–267. 2892843 10.1083/jcb.106.2.253 PMC2114972

[38] Lehr CM. Lectin-mediated drug delivery: the second generation of bioadhesives. J Control Release. 2000;65(1–2):19–29. 10699266 10.1016/s0168-3659(99)00228-x

[39] Balin BJ , Broadwell RD. Lectin-labeled membrane is transferred to the Golgi complex in mouse pituitary cells in vivo. J Histochem Cytochem. 1987;35(4):489–498. 2434560 10.1177/35.4.2434560

[40] Coelho LC , Silva PM , Lima VL , et al. Lectins, interconnecting proteins with biotechnological/pharmacological and therapeutic applications. Evid Based Complement Alternat Med. 2017;2017:1594074. 28367220 10.1155/2017/1594074 PMC5359455

[41] Freed DL. Do dietary lectins cause disease? BMJ. 1999;318(7190):1023–1024. 10205084 10.1136/bmj.318.7190.1023 PMC1115436

[42] Nachbar MS , Oppenheim JD. Lectins in the United States diet: a survey of lectins in commonly consumed foods and a review of the literature. Am J Clin Nutr. 1980;33(11):2338–2345. 7001881 10.1093/ajcn/33.11.2338

[43] Liener IE. Phytohemagglutinins: their nutritional significance. J Agric Food Chem. 1974;22(1):17–22. 4590577 10.1021/jf60191a031

[44] Zakharov A , Carchilan M , Stepurina T , Rotari V , Wilson K , Vaintraub I. A comparative study of the role of the major proteinases of germinated common bean (Phaseolus vulgaris L.) and soybean (Glycine max (L.) Merrill) seeds in the degradation of their storage proteins. J Exp Bot. 2004;55(406):2241–2249. 15333645 10.1093/jxb/erh247

[45] Pusztai A , Clarke EM , King TP. The nutritional toxicity of Phaseolus vulgaris lectins. Proc Nutr Soc. 1979;38(1):115–120. 461432 10.1079/pns19790015

[46] Banwell JG , Howard R , Cooper D , Costerton JW. Intestinal microbial flora after feeding phytohemagglutinin lectins (Phaseolus vulgaris) to rats. Appl Environ Microbiol. 1985;50(1):68–80. 4026292 10.1128/aem.50.1.68-80.1985 PMC238575

[47] Kik MJ , Rojer JM , Mouwen JM , Koninkx JF , van Dijk JE , van der Hage MH. The interaction between plant lectins and the small intestinal epithelium: a primary cause of intestinal disturbance. Vet Q. 1989;11(2):108–115. 2662569 10.1080/01652176.1989.9694207

[48] King TP , Pusztai A , Clarke EM. Kidney bean (Phaseolus vulgaris) lectin-induced lesions in the small intestine: 1. Light microscope studies. J Comp Pathol. 1980;90(4):585–595. 7276267 10.1016/0021-9975(80)90107-3

[49] Liu B , Cheng Y , Bian HJ , Bao JK. Molecular mechanisms of Polygonatum cyrtonema lectin-induced apoptosis and autophagy in cancer cells. Autophagy. 2009;5(2):253–255. 19139634 10.4161/auto.5.2.7561

[50] Dokladny K , Myers OB , Moseley PL. Heat shock response and autophagy--cooperation and control. Autophagy. 2015;11(2):200–213. 25714619 10.1080/15548627.2015.1009776 PMC4502786

[51] McCormick JJ , Dokladny K , Moseley PL , Kenny GP. Autophagy and heat: a potential role for heat therapy to improve autophagic function in health and disease. J Appl Physiol. 2021;130(1):1–9. 33119472 10.1152/japplphysiol.00542.2020

[52] Kaushik S , Cuervo AM. The coming of age of chaperone-mediated autophagy. Nat Rev Mol Cell Biol. 2018;19(6):365–381. 29626215 10.1038/s41580-018-0001-6 PMC6399518

[53] Chiang HL , Terlecky SR , Plant CP , Dice JF. A role for a 70-kilodalton heat shock protein in lysosomal degradation of intracellular proteins. Science. 1989;246(4928):382–385. 2799391 10.1126/science.2799391

[54] Dice JF. Peptide sequences that target cytosolic proteins for lysosomal proteolysis. Trends Biochem Sci. 1990;15(8):305–309. 2204156 10.1016/0968-0004(90)90019-8

[55] Dokladny K , Zuhl MN , Mandell M , et al. Regulatory coordination between two major intracellular homeostatic systems: heat shock response and autophagy. J Biol Chem. 2013;288(21):14959–14972. 23576438 10.1074/jbc.M113.462408 PMC3663517

[56] Sparvoli F , Faoro F , Daminati MG , Ceriotti A , Bollini R. Misfolding and aggregation of vacuolar glycoproteins in plant cells. Plant J. 2000;24(6):825–836. 11135116 10.1046/j.1365-313x.2000.00933.x

[57] Hickman S , Kulczycki A, Jr., Lynch RG , Kornfeld S. Studies of the mechanism of tunicamycin in hibition of IgA and IgE secretion by plasma cells. J Biol Chem. 1977;252(12):4402–4408. 325006

[58] Kuznetsov G , Chen LB , Nigam SK. Multiple molecular chaperones complex with misfolded large oligomeric glycoproteins in the endoplasmic reticulum. J Biol Chem. 1997;272(5):3057–3063. 9006956 10.1074/jbc.272.5.3057

[59] Pahl HL , Baeuerle PA. Endoplasmicreticulum-induced signal transduction and gene expression. Trends Cell Biol. 1997;7(2):50–55. 17708906 10.1016/S0962-8924(96)10050-7

[60] Ben-Zvi AP , Goloubinoff P. Review: mechanisms of disaggregation and refolding of stable protein aggregates by molecular chaperones. J Struct Biol. 2001;135(2):84–93. 11580258 10.1006/jsbi.2001.4352

[61] Liberek K , Lewandowska A , Zietkiewicz S. Chaperones in control of protein disaggregation. EMBO J. 2008;27(2):328–335. 18216875 10.1038/sj.emboj.7601970 PMC2234349

[62] Dobson CM. Principles of protein folding, misfolding and aggregation. Semin Cell Dev Biol. 2004;15(1):3–16. 15036202 10.1016/j.semcdb.2003.12.008

[63] Rieger TR , Morimoto RI , Hatzimanikatis V. Bistability explains threshold phenomena in protein aggregation both in vitro and in vivo. Biophys J. 2006;90(3):886–895. 16299080 10.1529/biophysj.105.066662 PMC1367113

[64] Bukau B , Weissman J , Horwich A. Molecular chaperones and protein quality control. Cell. 2006;125(3):443–451. 16678092 10.1016/j.cell.2006.04.014

[65] Lee S , Tsai FT. Molecular chaperones in protein quality control. J Biochem Mol Biol. 2005;38(3):259–265. 15943899 10.5483/bmbrep.2005.38.3.259

[66] Mayer MP , Bukau B. Hsp70 chaperones: cellular functions and molecular mechanism. Cell Mol Life Sci. 2005;62(6):670–684. 15770419 10.1007/s00018-004-4464-6 PMC2773841

[67] Afrazi A , Sodhi CP , Good M , et al. Intracellular heat shock protein-70 negatively regulates TLR4 signaling in the newborn intestinal epithelium. J Immunol. 2012;188(9):4543–4557. 22461698 10.4049/jimmunol.1103114 PMC3331906

[68] Musch MW , Ciancio MJ , Sarge K , Chang EB. Induction of heat shock protein 70 protects intestinal epithelial IEC-18 cells from oxidant and thermal injury. Am J Physiol. 1996;270(2):C429–436. 8779904 10.1152/ajpcell.1996.270.2.C429

[69] Musch MW , Sugi K , Straus D , Chang EB. Heat-shock protein 72 protects against oxidant-induced injury of barrier function of human colonic epithelial Caco2/bbe cells. Gastroenterology. 1999;117(1):115–122. 10381917 10.1016/s0016-5085(99)70557-3

[70] Dokladny K , Ye D , Kennedy JC , Moseley PL , Ma TY. Cellular and molecular mechanisms of heat stress-induced up-regulation of occludin protein expression: regulatory role of heat shock factor-1. Am J Pathol. 2008;172(3):659–670. 18276783 10.2353/ajpath.2008.070522 PMC2258255

[71] Ryan AJ , Flanagan SW , Moseley PL , Gisolfi CV. Acute heat stress protects rats against endotoxin shock. J Appl Physiol. 1992;73(4):1517–1522. 1447099 10.1152/jappl.1992.73.4.1517

[72] Mosser DD , Martin LH. Induced thermotolerance to apoptosis in a human T lymphocyte cell line. J Cell Physiol. 1992;151(3):561–570. 1295903 10.1002/jcp.1041510316

[73] Van Molle W , Wielockx B , Mahieu T , et al. HSP70 protects against TNF-induced lethal inflammatory shock. Immunity. 2002;16(5):685–695. 12049720 10.1016/s1074-7613(02)00310-2

[74] Gabai VL , Mabuchi K , Mosser DD , Sherman MY. Hsp72 and stress kinase c-jun N-terminal kinase regulate the bid-dependent pathway in tumor necrosis factor-induced apoptosis. Mol Cell Biol. 2002;22(10):3415–3424. 11971973 10.1128/MCB.22.10.3415-3424.2002 PMC133785

[75] Kluger MJ , Rudolph K , Soszynski D , et al. Effect of heat stress on LPS-induced fever and tumor necrosis factor. Am J Physiol. 1997;273(3):R858–863. 9321860 10.1152/ajpregu.1997.273.3.R858

[76] Dokladny K , Kozak A , Wachulec M , et al. Effect of heat stress on LPS-induced febrile response in D-galactosamine-sensitized rats. Am J Physiol Regul Integr Comp Physiol. 2001;280(2):R338–344. 11208560 10.1152/ajpregu.2001.280.2.R338

[77] Dokladny K , Lobb R , Wharton W , Ma TY , Moseley PL. LPS-induced cytokine levels are repressed by elevated expression of HSP70 in rats: possible role of NF-kappaB. Cell Stress Chaperones. 2010;15(2):153–163. 19551494 10.1007/s12192-009-0129-6 PMC2866987

